# Whole Black Rice Flour Improves the Physicochemical, Glycemic, and Sensory Properties of Cracker Snacks

**DOI:** 10.3390/foods13101503

**Published:** 2024-05-13

**Authors:** Alexandra Maria Uivarasan, Leonard Mihaly Cozmuta, Jasmina Lukinac, Marko Jukić, Gordana Šelo, Anca Peter, Camelia Nicula, Anca Mihaly Cozmuta

**Affiliations:** 1 Department of Chemistry-Biology, Technical University of Cluj Napoca, 430122 Baia Mare, Romania; uivarasan.al.alexand@student.utcluj.ro (A.M.U.); leonard.mihaly@cb.utcluj.ro (L.M.C.); anca.peter@cb.utcluj.ro (A.P.); camelia.nicula@cb.utcluj.ro (C.N.); 2 Faculty of Food Technology, Josip Juraj Strossmayer University of Osijek, 31000 Osijek, Croatia; jlukinac@ptfos.hr (J.L.); marko.jukic@ptfos.hr (M.J.);

**Keywords:** whole brown rice, black rice, cereals, blood glucose, gluten-free crackers, polyphenol-rich, glycemic peak

## Abstract

The present study describes the enhancement of the nutritional values of gluten-free rice crackers by adding whole black rice grain flour. The crackers were prepared by combining whole brown rice flour (WRF) and whole black rice flour (BRF) in ratios of 0% (WRC), 25% (25-BRC), 50% (50-BRC), 75% (75-BRC), and 100% (BRC). The resulting samples underwent in-vivo effects on postprandial blood glucose levels as well as physicochemical and sensory analysis. In comparison to WRC, the samples containing 100% added black rice flour presented higher nutritional qualities in terms of protein, by 16.61%, 8.64% for lipids, 5.61% for ash, 36.94% for crude fiber, 58.04% for total polyphenols, 95.49% for proanthocyanidins, and 88.07% for flavonoids. The addition of BRF had a suppressing effect on lightness (L*) and yellowness (b*), while redness (a*) increased. The results of the glycemic measurements confirmed that consumption of crackers made from brown or black whole-grain rice grain flour does not generate glycemic peaks above the limit of 30 mg/dL in baseline blood glucose levels. The results of developing rice crackers from black and brown flour blends showed promising physicochemical and nutritional properties and could provide a good alternative to wheat flour as a gluten-free product.

## 1. Introduction

Worldwide, people have been consuming cereal-based foods in a variety of forms, including breakfast cereals, bakery goods, pasta and noodles, snack foods, and others, as a staple source of energy and nutrients [1]. Due to their extended shelf life and high eating quality, cracker consumption is dramatically increasing [2]. As a result, crackers have emerged as one of the most popular snacks [3]. There are several types of crackers, such as water biscuits, puff biscuits, cream crackers, and saltine and soda crackers. These usually have minimal water content (15–25%) and laminated dough that bakes to a crunchy texture [4]. However, all supermarket crackers are generally made using wheat flour, and have a low content of nutrients, and a high glycemic index. Improving the nutritional profile of already-existing food products or developing new functional products are common goals of food producers, as they may also help in preventing or controlling certain metabolic diseases such as type 2 diabetes mellitus or celiac sprue [5,6].

In recent years, the consumption of foods with nutraceuticals and bioactive properties, especially fully pigmented rice grains, has gained importance [7]. The main cereal, rice (*Oryza sativa*), is consumed by humans almost solely as food. Brown rice, or dehusked raw rice, is coated in layers of bran, called testa, aleurone, and pericarp layers [8]. One variety of the rice species (*Oryza sativa* L.) is black rice, which is primarily grown in Asia and loaded with nutrients. Anthocyanin is responsible for the black color of the pericarp (outer part) of this particular rice kernel. This rice grain has minimal amounts of sugar, salt, and fat, and is free of gluten [9]. Black rice has a high fiber content, anthocyanins, antioxidants, vitamins B and E, iron, thiamine, magnesium, niacin, and phosphorus [9].

Some of the many distinctive qualities of whole rice grains are their hypoallergenic qualities, bland taste, and ease of digestion [10]. However, most of the proteins found in rice are highly hydrophobic, which means that even at a neutral pH, they resist swelling in water [10,11]. According to Juliano [11], the elastic properties of native wheat glutens, which are required to produce baked goods, are missing from rice proteins. Nevertheless, due to its low protein content and lack of gliadin, black and brown rice are a perfect food source for different types of consumers with low protein needs or celiac disease sprue (gluten intolerance) meals [12]. As such, it is typically used to improve the nutritional quality of food.

By fully replacing wheat flour with whole black rice flour, the objective of this study was to enhance the technological, sensory, and chemical properties of conventional crackers.

## 2. Materials and Methods

### 2.1. Raw Materials and Reagents

The two types of rice grains that were used to prepare the samples were obtained from two different feedstocks. Black rice (BRF) was purchased from Niavis, Pakistan, while brown rice (WRF) was purchased from Eco, Thailand. The grains were inspected for foreign materials and broken particles, then ground using an IKA Labortechnik MF 10 grinder (IKA-Werke GmbH and Co., KG, Staufen im Breisgau, Germany) and passed through a 1 mm sieve. Black rice flour was incorporated into three different flour mixtures: 25% (25-BRF), 50% (50-BRF), and 75% (75-BRF) for the preparation of rice flour crackers (Table 1).

Whey protein powder (whey protein concentrate, 80 g protein/100 g product, SFD, Opole, Poland), xanthan gum powder (Nutrimedica, Zagreb, Croatia), baking powder (Dr. Oetker, Bielefeld, Germany), sugar (crystal, Viro, Zagreb, Croatia), salt (refined and iodized, Tuzlanska So, Tuzla, Bosnia), margarin (Zvijezda, Zagreb, Croatia), raw glucose (Feleacul, Cluj, Roamania) and Croco crackers (salted crackers, Croco, Onesti, Romania) were purchased from a local supermarket. Analytical grade products that were acquired from Merck (Rahway, NJ, USA) included hydrochloric acid (HCl), sodium hydroxide (NaOH), ethanol, acetic acid, iodine solution, methanol, hexane, sodium carbonate (Na_2_CO_3_), gallic acid, diethyl ether, and ethyl acetate. Sodium nitrite (NaNO_2_) was obtained from Gram Mol (Zagreb, Croatia). Standards for UHPLC analysis (−)-epicatechin; epicatechin gallate; gallocatechin gallate; (+)-catechin hydrate; phenolic acids (syringic, ellagic, p-coumaric, o-coumaric, caffeic, ferulic, and p-hydroxybenzoic); resveratrol, rutin hydrate, and kaempferol were purchased from Sigma Aldrich (Saint Louis, MO, USA). Glacial acetic acid (99.5%) was purchased from Macron Fine Chemicals (Gliwice, Poland); vanillic acid, 3,4-dihydroxybenzoic acids, and quercetin were purchased from Acros Organics (Geel, Belgium); and n-hexane was purchased from Carlo Erba Reagents GmbH (Emmendingen, Germany). HPLC grade acetonitrile was from Fisher Chemical (Loughborough, UK) and ultra-gradient grade methanol from J.T. Baker (Arnhem, The Netherlands); procyanidins B1 and B2, oenin chloride, myrtillin chloride, kuromanin chloride, petunidin chloride, callistephin chloride, and peonidin-3-O-glucoside chloride were purchased from Extrasynthese (Genay, France); aluminum chloride hexahydrate from Alfa Aesar GmbH & Co KG (Kandel, Germany); and 1-butanol from Fisher Scientific (Loughborough, UK). Ferrous sulfate was purchased from Kemika (Zagreb, Croatia).

### 2.2. Preparation of Rice Crackers

**Figure 1:**
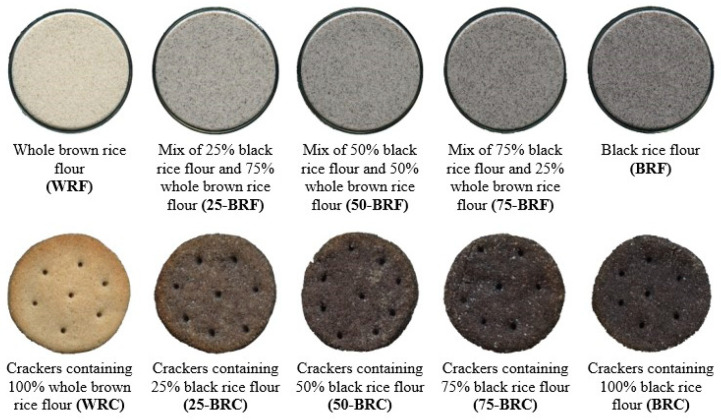
Appearance of whole brown rice flour, black rice flour, their mixtures, and corresponding crackers.

Five types of rice crackers were prepared using the method suggested by Nakov et al. [13] (Table 1), of which two were made of brown rice flour (WRC) and black rice flour (BRC), and the other three by replacing the whole brown rice flour in ratios of 25% substitution with black rice flour (25-BRC), 50% (50-BRC) substitution, and 75% substitution (75-BRC) (Figure 1). Two types of dough were meticulously prepared: a basic dough and a fatty dough (ingredients are detailed in the Supplementary Materials, Table S1), which were subsequently combined to form the final dough. For preparing the basic dough, the ingredients were mixed for 7 min (Biovita MB-1500 PRO, Cluj Napoca, Romania) at 2200 rpm until a homogenous dough was developed. For the fatty dough, the ingredients were manually mixed until a cohesive spherical shape was achieved. The prepared doughs were integrated, resulting in a homogeneous mixture. The combined dough was then passed through a laminator (Prismafood Solutions, Pordenone, Italy) and cut into uniform pieces with a diameter of 4 cm. The dough pieces were arranged on a baking tray (30 × 40 cm) and transferred to an oven (Piron, Milano, Italy) for baking at 190 °C for 12 min. Following baking, the crackers were allowed to cool naturally at room temperature (25 °C) for 30 min before undergoing further analysis.

### 2.3. Characterization of Rice Crackers

#### 2.3.1. Proximate Analysis

The following parameters of the prepared rice crackers were determined: total lipids (AOAC 920.39.C, 1995) [14], ash content (AOAC 936.07) [15], and total protein content (AOAC 945.18-B, 1995) [16] (the conversion factor of nitrogen to protein was 6.25), crude fiber (Commission Regulation No. 152/2009) [17], moisture content (AOAC 925.10, Section 2.2 in Supplementary Materials) [18], and water activity (aw) (Section 2.3 in Supplementary Materials).

#### 2.3.2. Carbohydrates

Based on Equation (1), the carbohydrate content was calculated:

(1) $$\;Carbohydrates\left(g/100g\right)=100-(protein+fat+ash+fiber)$$

#### 2.3.3. Dimensions

The dimensions of the samples were measured following the guidelines outlined in AACC International Method 10-50.05 [19]. The measurement process involved the following steps: six crackers were aligned side by side, and their total width was measured. Subsequently, each cracker was rotated by 90° and measured individually. The average width of each cracker (W, cm) was then calculated by dividing the total width by six. The total height of six crackers was measured when the samples were stacked on top of one another. The crackers were then rearranged at random, and the height was measured once more. The average height of each cracker (H, cm) was calculated by dividing the height of six crackers by six. For each batch, six sample crackers were measured. The spread factor was calculated as width/thickness multiplied by 10.

#### 2.3.4. Energy

According to Annex XIV of EU Regulation No. 1169/2011 [20], the energy supplied by crackers was calculated using the formula:

(2) $$Energyvalue\left(kcal/100g\right)=4\cdot carbohydrate\left(\%\right)+4\cdot protein\left(\%\right)+2\cdot fiber\left(\%\right)+9\cdot fat(\%)$$

#### 2.3.5. Total Starch and Amylose Content

The AACC 76–13 method [21] was used to measure the total starch content of the rice crackers. The method is based on the hydrolysis of starch by α-amylase into maltodextrins, which are then further broken down into D-glucose by amyloglucosidase. Glucose is oxidized to D-gluconate and hydrogen peroxide, which is further decomposed by peroxidase with the formation of quinonimine. The intensity of the colored compound was spectrophotometrically quantified at 510 nm (Perkin Elmer Lamba 35 UV-VIS).

The amount of amylose in rice flours was measured using the iodine-binding method [22]. A volume of 1 mL of 9 N NaOH and 1 mL of 95% ethanol were thoroughly mixed with 100 mg of sample. The starch was gelatinized by heating the solution in a boiling water bath for 10 min, after which it was allowed to cool to room temperature. A volume of 5 mL of the gelatinized starch solution was added to a 100 mL volumetric flask along with 1 mL of 1 N acetic acid and 2 mL of iodine solution. The mixture was then diluted with distilled water to a final volume of 100 mL and homogenized for 20 min. The absorbance was measured at 620 nm (Perkin Elmer Lambda 35 UV-VIS spectrophotometer). The amylose content of the sample was measured using a standard curve based on rice amylose.

#### 2.3.6. Polyphenol Extraction and Measurement

The rice cracker samples were defatted using the method that Shamanin et al. [23] proposed, adapted to our work. Briefly, hexane was combined with the crackers sample (1:5 *w*/*v*), stirred for 10 min at 200 rpm, and centrifuged for 5 min at 2500× *g*. The supernatant was removed, and the solid phase was mixed again with hexane in respect of the initial ratio and centrifuged. After three extraction rounds, the defatted samples were dried on filter paper in a fume hood for 12 h.

The method proposed by Jang and Xu [24] was followed to extract the *free phenolic compounds*. Using 30 mL of 80% aqueous methanol and ultrasonication (Bandelin Sonorex Super RK, BANDELIN electronic GmbH and Co. Germany, KG, 35 KHz, 80 W) for 25 min at 25 °C, an accurately weighed 5 g of defatted sample was extracted. The mixture was centrifuged at 2500× *g* for 5 min, and the solid phase underwent two additional extractions that were identical to the first one. The supernatants containing free phenolic compounds were combined and subjected to analysis.

*The total phenolic compounds* (TPC) were extracted by mixing 5 g of defatted sample with 50 mL of 95% ethanol at 200 rpm at room temperature over a period of six days [25]. All extractions were performed in two replicates. To measure the polyphenolic content, a volume of 0.5 mL of 20% Folin-Ciocalteu reagent was added to a volume of 2 mL of extract. The mixture was then allowed to react for 5 min, after which 0.5 mL of saturated Na_2_CO_3_ solution and 5 mL of deionized water were added and kept in the dark for 2 h. The absorbance of the solution was measured at 765 nm (Perkin Elmer Lambda 35 UV-VIS spectrophotometer). The phenolic compounds were quantified using the gallic acid-based calibration curve, and the results were expressed as mg gallic acid equivalent (GAE)/100 g of sample.

The difference between the contents of total and free phenolic compounds was calculated as *bound phenolic content*.

#### 2.3.7. Total Proanthocyanidin Content (TPA)

Based on the acid-butanol reaction with black rice extract, TPAs were determined according to the method described by Škerget et al. [26] with minor modifications. Briefly, 0.5 mL of the rice cracker extract was added to 5 mL of an iron (II) sulphate heptahydrate solution prepared by dissolving 77 mg of FeSO_4_·7H_2_O in 500 mL of HCl-butanol solution (2:3, *v*/*v*). The mixture was stirred and incubated in a water bath at 95 °C. After 15 min, the mixture was cooled under water, and the absorbance was measured at 540 nm against a blank sample containing distilled water in place of the sample. The TPA content was calculated according to the molar extinction coefficient and the molar weight of cyanidin, and the results were expressed as mg per g of the dry basis of the sample (mg/g).

#### 2.3.8. Total Flavonoid Content (TFV)

TFV (total flavonoid content) was measured using the spectrophotometric method (Perkin Elmer Lambda 35 UV-VIS spectrophotometer) with aluminum chloride, according to Marinova et al. [27], with minor modifications. More exactly, a volume of 0.5 mL of rice cracker extract was mixed with 2 mL of distilled water in a volumetric flask. Then, 0.15 mL of a 5% (*w*/*v*) NaNO_2_ solution was added, and after 5 min, 0.15 mL of a 10% (*w*/*v*) AlCl_3_ solution was added as well. After exactly 6 min, 1 mL of 1 M NaOH was added, and the mixture was diluted with 1.2 mL of distilled water. The absorbance was measured at 510 nm against a blank. The results were expressed as (+)- mg catechin equivalent per g of dry basis of sample (mgCE/g). Each sample was analyzed in three replicates.

#### 2.3.9. Determination of Individual Phenolic Compounds and Anthocyanins

Ultra-high performance liquid chromatography (UHPLC Nexera XR, Shimadzu, Tokyo, Japan) was used to quantify the individual phenolic compounds (phenolic acids and anthocyanins). Separation was performed with a reversed-phase Kinetex^®^ C18 core-shell column (100 × 4.6 mm, 2.6 µm, Phenomenex, Torrance, CA, USA), and a photodiode detector (PDA) was used. Prior to UHPLC analyses, samples are filtered through 0.45-µm membranes. The data obtained was processed using LabSolutions 5.87 software. The determination of phenolic acids was performed following the protocol published by Martinović et al. [28] using a linear gradient of two phases: A: methanol/acetonitrile (50:50, *v*/*v*) and B: 1.0% acetic acid in water (*v*/*v*). With a flow rate of 1 mL/min and at 30 °C, a linear gradient was performed from 5% to 30% B in 25 min, from 30% to 40% B in 10 min, from 40% to 48% B in 5 min, from 48% to 70% B in 10 min, from 70% to 100% B in 5 min, isocratic at 100% B for 5 min, followed by a return to baseline conditions (10 min) and column equilibration (10 min). The sample injection volume was 20 µL.

The determination of anthocyanins by the UHPLC method was performed according to Martinović et al. [28]. Two mobile phases were used: A: water/formic acid/acetonitrile (87:10:3, *v*/*v*/*v*) and B: water/formic acid/acetonitrile (40:10:50, *v*/*v*/*v*) with a gradient program; 10 min from 10 to 25% mobile phase B, 5 min from 25 to 31% mobile phase B, 5 min from 31 to 40% mobile phase B, 10 min from 40 to 50% mobile phase B, 10 min from 50 to 100% mobile phase B, and 10 min from 100 to 10% mobile phase B. The injection volume of the sample was 20 µL at a flow rate of 0.8 mL/min. By comparing the UV-Vis spectra and retention times of authentic standards analyzed under the same conditions, individual phenolic compounds were detected and quantified using the calibration curves generated with the external standards. Anthocyanins were determined at 513–531 nm. Phenolic acids were determined at 252–322 nm.

#### 2.3.10. Color Analysis

The color of the rice flour crackers was assessed according to the CIEL*a*b* color system (which characterizes colors based on parameters L*, a*, and b*) using a Croma Meter (Konica Minolta CR-400, Tokyo, Japan). The luminance or brightness of the sample (L*) represents the brightness on a scale from 0 (black) to 100 (white). The range of the chromatic components a* and b* is between −128 and 127. The green-red and blue-yellow axes of the color space are denoted by the parameters a* and b*, respectively. The instrument was calibrated before each measurement against a standard white tile CR-A43 (L* = 92.46; a* = −0.86; b* = −0.16), with measurements taken at five different places of the crackers, at a temperature of 25 °C.

The total color difference (∆E) between the control and sample crackers was calculated according to Equation (3) [29], which represents the Euclidean distance between two points in the CIEL*a*b* color space. From each batch, two representative crackers were evaluated.

(3) $$\Delta E=\sqrt{\Delta L^{2}+\Delta a^{2}+\Delta b^{2}}$$

where ∆L represents brightness difference, ∆a represents redness difference, and ∆b represents yellowness difference;

Browning index (BI) was calculated using Equation (4) [2]:

(4) $$BI=\frac{\left[100\left(\frac{a^{*}+1.75\cdot L^{*}}{5.645\cdot L^{*}+a^{*}-3.012\cdot b^{*}}-0.31\right)\right]}{0.17}$$

where L*, a*, and b* are the chromatic components of the CIEL*a*b* color system.

Chroma (C*) was calculated according to the equation proposed by Kyriakoudi et al. [30]:

(5) $$C^{*}=\sqrt{{\left(a^{*}\right)}^{2}+{\left(b^{*}\right)}^{2}}$$

where a* and b* are the color components of the CIEL*a*b* system. Chroma refers to the intensity or purity of a color, indicating how vivid or dull it appears. A higher C* value indicates a more intense or saturated color, while a lower value suggests a less saturated color.

#### 2.3.11. Texture Profile Analysis

The texture profile analysis of the investigated crackers was determined by using a TA.XT2i Texture Analyzer (Stable Microsystems Ltd., Surrey, UK). A knife blade moved toward the cracker located between the two lower supports, with a distance of 25 mm between each other. The compression test was used for the analysis. The textural attributes measured were the following: The textural attributes measured were the following: Hardness (N), presented as the peak force to snap the cracker; Peak count, as a measure of crispiness, reflecting the number of texture peaks observed during the texture analysis, where a higher count of peaks typically corresponds to a more pronounced crunch sensation, indicating greater crispness; Work of shear (Ns), which indicates the average of the forces required to cut through the sample and serves as a measure of the energy required to deform the cracker sample during the texture analysis, and Distance to maximum peak (mm), which refers to the distance between the initial compression point and the peak force observed during the texture analysis [31], with shorter distances suggesting greater fragility or brittleness. The specific parameters used for testing included a pre-test speed of 3.00 mm/s, a post-test speed of 10.00 mm/s, test mode set to compression, and trigger force set at 0.049 N.

The test was performed in 10 replicates for each sample, and the results were expressed as mean ± standard deviations.

#### 2.3.12. Blood Glucose Level Measurement

To highlight the influence of consumption of whole rice flour crackers on the variation of postprandial blood glucose, three experiments with WRC, BRC, and “Croco” crackers were conducted according to the protocol described by Saito et al. [32], adapted to our work. Croco crackers were chosen to be used as a reference for traditional supermarket snacks. The study was approved by the Ethics Committee of the Technical University of Cluj-Napoca, Romania (nr. 573/11.01.2024). A number of 50 clinically healthy subjects (26 men and 24 women) with normal body mass indexes (BMI’s) (between 19.30 ± 2.02 kg/m^2^ and 24.50 ± 2.70 kg/m^2^, without being under the influence of certain medications) and an average age of 42.90 ± 15.50 years (minimum 19 years, maximum 73 years) participated in the study. Prior to conducting the experiment, participants were informed regarding the aim of the study and asked to sign a consent form, indicating their agreement to the terms and conditions of the experiment. The day before the experiment, subjects were instructed not to consume food and beverages (except water) after 8 PM, and the next day, blood glucose measurements (Accu-Chek Active Glucometer, Roche Diabetes Care, Inc. Basel, Switzerland) were conducted on an empty stomach at 8:00 AM, after which each subject consumed 49.73 g of brown rice crackers (containing 35 g of available carbohydrates for serving) and 100 mL of water.

Blood glucose was collected from the fingertip at five time points: baseline, 30, 60, 90, and 120 min after the ingestion of the crackers. The experiment was repeated 3 times, evaluating the influence of the consumption of black and brown rice crackers and conventional crackers on blood glucose variations. The amount of 58.30 g of Croco crackers (containing the same amount of available carbohydrates as the rice crackers) served as a control. The diet of each subject was kept constant the day before the tests.

#### 2.3.13. Sensory Study Protocol

A group of 100 panelists (49 men and 51 women) with an average age of 42.47 ± 15.01 years (minimum 19 years, maximum 72 years) previously instructed and accustomed to crackers participated in the sensory analysis of the examined samples within 3 h of baking in daylight and at room temperature (21 °C), following the methods described by Han et al. [33] and De Kock et al. [34]. The research, approved by the Ethics Committee of the Technical University of Cluj-Napoca, Romania (nr. 532/19.04.2023), followed the ethical guidelines of the 1975 Declaration of Helsinki, revised in 2013. The participants provided their written consent and were informed about the purpose and objective of the study before the sensory investigation. They were also instructed to familiarize themselves with the descriptive lexicon of crackers. Samples of each cracker were coded, arranged at random on the plate, and presented to the panelists for tasting in three evaluation sessions 30 min apart. In between sessions, the panelists rinsed their mouths with room-temperature water to prevent the carryover effect. Panelists were seated in separate testing booths to evaluate the product. A nine-point hedonic scale was used to rate the shape, color, texture, smell, taste, and overall acceptability (scored 1 to 9, 1—dislike extremely to 9—like extremely) of the products. The average of the individual scores was used to calculate the overall score for each attribute.

### 2.4. Statistic Analysis

In every experiment, a minimum of three replications were conducted, and the results were expressed as the mean ± standard deviation. Statistical analyses were performed using the one-way analysis of variance (ANOVA) test, with a significance level set at *p* < 0.05. Principal component analysis (PCA) was performed using Statistics 7.0 software. Additionally, the Pearson correlation coefficient was utilized to analyze the data and determine the relationship between the important variables, with a significance level set at *p* < 0.01.

## 3. Results and Discussion

### 3.1. Proximate Composition of Rice Crackers

The visual representation of the rice crackers under investigation is displayed in Figure 1, while Table 2 provides a brief overview of their distinctive features. The moisture content of the crackers increased by up to 10.34% depending on the ratio, due in large part to the replacement of WRF with BRF. The results matched the findings published by Giannoutsos et al. [35], who reported a range of moisture percentages from 2% to 5.6%. Subsequent analyses of all samples revealed higher ash contents. The maximum ash content was achieved in BRC, with a value of 4.64 ± 0.08%, followed by WRC (4.38 ± 0.02%), 25-BRC (4.33 ± 0.14%), 50-BRC (4.32 ± 0.10%), and 75-BRC (4.15 ± 0.09%). The total lipid, protein, and crude fiber content of the crackers increased significantly with an increasing BRF substitution ratio. Substituting 50% BRF increased the protein content of the crackers by 10.76%. The results were similar to those obtained by Melini et al. [36] in terms of protein content. The lipid content showed the highest value in BRC (13.90 ± 0.30%), higher by 8.63% compared to WRC. Crude fiber showed an increase of 36.94% in BRC (*p* < 0.01) compared with WRC, while the carbohydrate content decreased by 6.82%. No significant differences (*p* > 0.05) were obtained between the carbohydrate content and energy provided by the crackers.

### 3.2. Color Analysis of Rice Crackers

Color was reported to have a direct impact on consumer perception and acceptability of crackers [4]. Extensive research has demonstrated that food color not only influences taste but also affects flavor intensity [35]. The color of the crackers was influenced by the content of yellow and red pigments in the black rice flour, namely flavonoids and anthocyanins, as well as the Maillard and caramelization reactions that occur during the baking process. According to the CIEL*a*b* analysis, the addition of black rice flour has a suppressing effect on the lightness (L*) of the crackers, giving them a darker shade (Table 3, Figure 1). For all crackers, the brightness decreased as the proportion of black rice flour (BRF) increased. The highest value of the lightness parameter, with L* = 70.75 ± 0.52 on the cracker surface and L* = 76.58 ± 0.21 in the crushed sample, was observed for the reference cracker samples (WRC). Experimental findings show that the addition of 25% black rice flour leads to a decrease of 38.31% in lightness on the surface and 38.87% in the crushed sample. In contrast, when the crackers are exclusively composed of black rice flour (BRC), the lightness diminishes significantly by 60.57% on the surface and 65.60% in the crushed sample. A similar trend was observed for the substitution of wheat with barley flour in biscuits [37] and the enrichment of wheat crackers with pulse flours [4].

**Figure 2:**
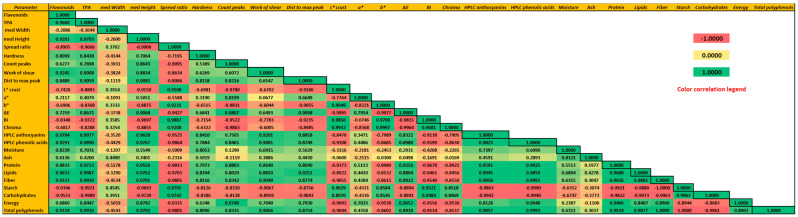
Pearson correlations between physico-chemical, textural, and chemical parameters of the analyzed crackers is TPA—total proanthocyanidin content.

The highest b* value was observed in control crackers, in which no anthocyanins were present. The increased replacement of whole brown rice flour with black rice flour resulted in higher values for a* (redness) and significantly lower values for b* (*p* < 0.05) (yellowness) compared to the control (WRC). A similar trend was observed by Szkudlarz et al. [38] in cookies enriched with polyphenols from grape pomace. Similarly, Saeed et al. [39] observed decreased L* and b* values and increased a* values for biscuits containing black gram (*Vigna mungo*) flour. Lower values of b* (larger blue content) can be explained by the higher content of total phenolic compounds identified in the samples containing 25%, 50%, 75%, and 100% black rice flour. The chromatic attribute C* (chroma or saturation) was significantly different (*p* < 0.01) between all analyzed crackers and the control, with variations from 5.19 ± 0.22 for BRC on the cracker surface to 26.30 ± 0.45 for WRC. A highly positive Pearson correlation (r = 0.99) between C* and L* is observed (Figure 2). WRC was identified with the highest C* value, denoting a more vivid color. Similar results were also obtained by Pasqualone et al. [40] for anthocyanin-enriched biscuits. The ΔΕ values showed an increase with the increasing degree of substitution of brown rice flour. For all samples, ΔΕ values showed that color differences were perceptible to the human eye (ΔΕ > 3). These results strongly support the assumption that the color of crackers is significantly influenced by the type of flour used, particularly in relation to the amount of flour used. Higher degrees of substitution appear to have a stronger effect. Browning index (BI) values were negatively correlated (r = −0.93) with proanthocyanidin and total phenolic content (r = −0.95). The crackers enriched with BRF showed significantly lower BI (*p* < 0.01) than the control sample. The lowest BI value (by 61.12%) was identified in BRC. The effect of lowering BI may be attributed to the antioxidant capacity of the phenolic compounds identified in the analyzed crackers. The main processes that give crust and crumb their dark color are the formation of melanoidins, the caramelization of sugars, and the thermal degradation of anthocyanins [41].

The type and proportion of the reducing sugars and amino acids involved appear to have an impact on the non-enzymatic browning reaction, which is responsible for the formation of color [41,42]. Higher protein content in the BRC contributes to higher production of melanoidins. The distinctive brown color of the crust layer is attributed to the formation of Maillard reaction products (MRPs), such as hydroxymethylfurfural (HMF) and melanoidins [43]. Additionally, brown compounds are formed as a result of sugar pyrolysis. The reduction in the hue values of a* and b* in the crust along with the rise in the ratio of BRF indicates an intense hydroxylation process of the B-rings of anthocyanins [44]. An increased degree of hydroxylation in the *B*-ring would shorten the half-life of the anthocyanins, while acylation appears to extend their half-life and slow their degradation process under heat treatment, in comparison to nonacylated derivatives [44]. According to Goto et al. [45], acylation of the molecules shields them from hydration, which increases anthocyanins stability.

Given the intense heat treatment crackers receive during baking, it is reasonable to assume that the color of the crust and crumb will change as the BRF ratio rises. No data in the literature regarding crackers made from mixes of pigmented and non-pigmented rice flour was found regarding the variations of CIEL*a*b* parameters.

### 3.3. Physical Properties and Texture Profile Analysis

The physical and textural properties of developed rice crackers are listed in Table 4.

No statistically significant differences between the sample diameters were found. By adding 25% BRF, the thickness of the crackers increased by 8.77% in 25-BRC and by 18.32% in BRC, as compared to standard. Spread ratio is regarded as one of the most crucial cracker quality parameters, being correlated with texture, bite, grain finesse, and overall mouth feel. A high spread ratio is preferred in the case of crackers [31]. The spread factor of developed rice crackers varied between 6.15 ± 0.23 in BRC and 7.53 ± 0.22 in WRC. The spread ratio showed a strong negative correlation (r = −0.98) with the protein content of the crackers, suggesting a higher protein content for the BRF replacement samples. Strong negative correlations were also found between spread ratio and count peaks, respectively, for shear (r = −0.91; r = −0.86). A higher concentration of BRF decreases the number of available hydrophilic sites for limited free water in dough. Islam et al. [46] reported that the formation of aggregates leads to an increase in the number of hydrophilic sites in composite flour.

During dough mixing, free water quickly partitions into hydrophilic sites, increasing dough viscosity and limiting cracker spread [47]. The decrease in spread ratio with the addition of BRF in the current study may also result from the increase in protein-rice content. When other ingredients absorb the water, the spread ratio is subsequently reduced [47]. Similar findings were reported by Quadri et al. [31] for the spread ratio in wheat-based crackers incorporated with brown rice flour and carboxymethyl cellulose. No significant statistical differences were found in the textural parameters of the samples (Table 4), suggesting that the addition of BRF does not affect texture.

### 3.4. Total Phenolic Composition of Rice Crackers

Table 5 shows the total phenolic compounds (TPC) present in the rice crackers. Because of their anti-inflammatory, anti-radical, and antioxidant properties, these substances contribute to human health protection [39]. Results showed that BRC was linked to higher total polyphenol concentrations by 58.04%, 34.60% free polyphenols, and 73.95% bound polyphenols.

The results exceeded those reported by Bolea et al. [48], who identified concentrations of 162.21 ± 0.39 mg GAE/100 g on appetizer biscuits based on black rice flour. Table 5 shows that the total flavonoid content (TFV) of the samples ranges from 309.31 ± 22.18 µg/g in WRC to 2592.07 ± 89.49 µg/g in BRC. Significant variations (*p* < 0.01) of the TFV were identified with the increase of BRF added to the composition of the samples. The results indicated that the addition of 25% BRF increased TFV by 49.00%, while the TFV was 88.07% higher in BRC than in control crackers. A comparable pattern was noted for the total proanthocyanidin content (TPA), with variations from 38.58 ± 0.29 µg/g in WRC to 854.75 ± 17.21 µg/g in BRC. Furthermore, as mentioned in Section 3.2, there is a strong negative correlation (r = −0.84) between TPA and the colorimetric parameter b* with increasing BRF concentration. This results in a tendency to suppress b*, which gives the crackers a bluer hue. According to earlier research [48,49], foods containing pigmented cereal varieties generally had much higher flavonoids and proanthocyanidin contents than foods containing non-pigmented cereal flour. The work of Pasqualone et al. [50] on the production of purple wheat (*Triticum aestivum*) functional biscuits reported values of 13.86 ± 0.27 mg Cy-3-Glu/kg for TPA and 2.58 ± 0.02 mg ferulic acid/g for TPC. Saeed et al. [39] found a TFV content of 93.36% higher than the control (wheat flour biscuits) in biscuits enriched with 20% black gram (*Vigna mungo*) flour, confirming the findings of this study.

### 3.5. Individual Phenolic Content

The content of individual phenolics in the analyzed cracker samples is displayed in Table 6. Anthocyanins are a subgroup of flavonoids mainly found in the pericarp, seed coat, and aleurone layer of rice. They are very sensitive to changes under the influence of light, oxygen, temperature fluctuations, and pH. There were significant differences (*p* < 0.05) between the content of individual anthocyanins in the extracts of the BRF-enriched crackers. This is to be expected, since black rice is a rich source of phenolic compounds [9]. Cyanidin 3-O-glucoside chloride (C3G) and peonidin-3-O-glucoside (P3G) chloride were detected in the largest amounts, with variations from 42.33 ± 1.55 µg/g (25-BRC) to 221.58 ± 2.07 µg/g (BRC) for C3G and between 23.27 ± 0.68 µg/g (25-BRC) to 120.25 ± 5.21 µg/g (BRC) for P3G, respectively. Oenin chloride and petunidin chloride were quantified in lower concentrations. Literature data shows that, due to their antioxidant activities and anti-inflammatory properties, C3G and P3G could potentially act as inhibitors of an inflammatory cytokine that is crucial for treating metabolic diseases linked to obesity [51,52]. Myrtillin chloride and callistephin chloride were not identified in any of the analyzed samples, while C3G, P3G, oenin chloride, and petunidin chloride were not detected in the control (WRC).

In the black rice cracker substitutes, the predominant phenolic acids identified were 3,4-dihydroxybenzoic acid and vanillic acid. 3,4-dihydroxybenzoic acid was not found in WRC but presented a concentration of 223.41 ± 1.28 µg/g in BRC. Vanillic acid reported values up to 98.90% higher in BRC as compared to WRC (*p* < 0.05). The concentration of ellagic acid was found to be 91.75% higher in the 25-BRF. Extensive research in vitro and in vivo [53,54] has confirmed that ellagic acid possesses antihyperlipidemic and antihyperglycemic properties in addition to its antioxidant and anti-inflammatory effects [55]. The p-hydroxybenzoic acid, procyanidin B2, and gallocatechin gallate could not be quantified in any of the samples. Most phenolic acids are found in soluble-conjugated or insoluble-bound forms. For soluble-free phenolic acids, p-coumaric acid, ferulic acid, sinapic acid, and ellagic acid were detectable in all the cracker samples, among which ferulic and ellagic acids were the most abundant. However, WRC outlined significantly higher levels (*p* < 0.05) of syringic (3.21 ± 0.13 µg/g), p-coumaric (1.45 ± 0.12 µg/g), ferulic (6.15 ± 0.16 µg/g), and sinapic acids (0.62 ± 0.06 µg/g) as compared to BRC. In rice, these phenolic acids, especially ferulic acid, can be bound to either the insoluble fiber that forms the cell walls or they can be ester linked to the sterols part of *y*-oryzanol in rice bran oil [56]. The color parameters L*, b*, and BI of the rice flour crackers presented negative correlations with TFC, TFV, TPA, and individual phenolics (Figure 2). The strong negative correlations between the color parameters and the anthocyanins suggest that pigmentation is related to the accumulation of anthocyanins from the rice flour in the composition of the crackers. These results are consistent with those published by Shao et al. [49] on the phenolic composition of brown, red, and black rice (*Oryza sativa*) cultivars.

### 3.6. Principal Component Analysis (PCA) of Data

**Figure 3:**
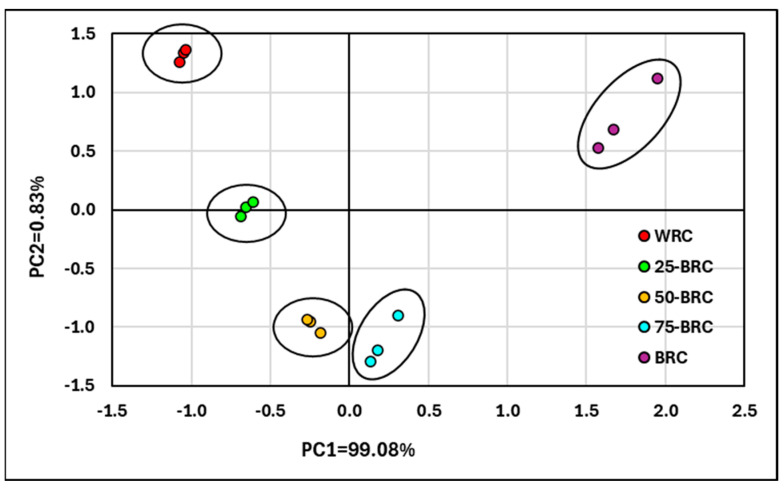
Principal component analysis (PCA) of 43 parameters in nutritional cracker preparation. Where WRC—crackers made of 100% whole brown rice flour; BRC—crackers made of 100% black rice flour; 25-BRC—crackers made of 25% black rice flour and 75% whole brown rice flour; 50-BRC—crackers made of 50% black rice flour and 50% whole brown rice flour; 75-BRC—crackers made of 75% black rice flour and 25% whole brown rice flour.

In the PCA data analysis (Figure 3), three data sets were used for each cracker type, for which (for the same sample) all 43 parameters were recorded according to Table 2, Table 3, Table 4, Table 5 and Table 6 (including compositional characteristics, color attributes, texture properties, and characterization regarding the content of phenolic compounds, flavonoids, and anthocyanins).

The top five principal components explain more than 99.99% of the variant. The first component explains 99.08%, and the second component explains 0.83% of the data variability. These results show that predominantly all compositional, structural, color, and content properties can be attributed to differences between WRC and BRC base raw materials.

Figure 3 shows the graphical dependence between the first two principal components. The grouping of points according to the composition of each type of cracker is highlighted. As BRF content increases, PC1 shifts from negative to positive values. Changing the composition of cookies by using WRF and BRF mixtures results in a decrease in PC2 values. Analysis of the data suggests that PCA analysis could also be used as a discriminative analysis in estimating the proportion of mixtures of the two types of rice flour in cookies.

### 3.7. Impact of the Consumption of Rice Flour-Based Crackers on Postprandial Blood Glucose Levels

**Figure 4:**
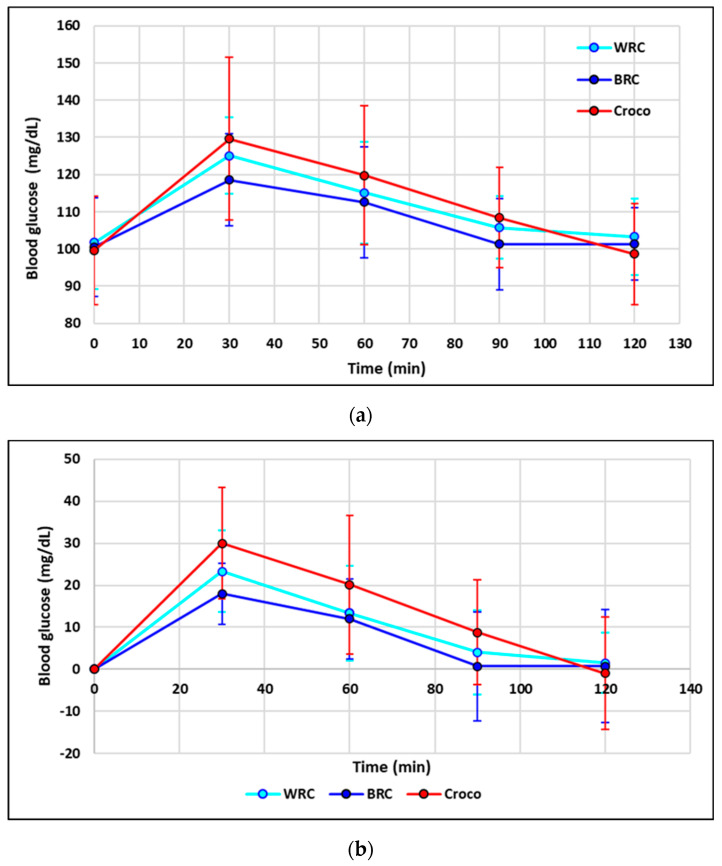
(**a**). The variation of average blood glucose levels, depending on time and type of product consumed, where the values at time t = 0 min correspond to the blood sugar values before consuming the tested products and the subsequent times 30, 60, 90, and 120 min represent the time elapsed after consumption of the products. (**b**). The variation of average blood glucose level increases depending on time and type of product consumed, where the values at time t = 0 min correspond to the blood sugar values before consuming the tested products, and the subsequent times 30, 60, 90, and 120 min represent the time elapsed after consumption of the products.

Table S2 presents the glycemic values corresponding to the consumption of the 3 products (WRC, BRC, and Croco crackers) as an average of the results. The standard deviations of the values ranged from 1.60 to 19.54. An ANOVA analysis was used to compare the values obtained, and a Tukey test was performed (for a statistical significance level of *p* < 0.05). The comparison was carried out in two variants: comparing the average blood glucose values at different points in time (for the same product consumed) to highlight increasing or decreasing effects; in this case, comparing the values per line. The letters a, b, and c were used. Blood glucose levels corresponding to the consumption of different products were tested for the same time values. In this case, the numbers 1, 2, and 3 were used to compare the column values. The application of the Tukey test did not reveal any statistically significant differences, which can be explained by the high variability of the data (blood sugar values). As shown in Figure 4a, in all cases, a peak in blood glucose is observed 30 min after consumption of the sample, followed by a decline in values up to 120 min. In the case of Croco cracker consumption, a glycemic maximum was identified at 30 min and a minimum at 120 min. The variations in the data corresponding to the consumption of the two types of brown and black rice flour crackers are roughly similar, with the observation that blood glucose levels are slightly higher when consuming brown rice flour crackers 30 min after consumption. However, the analysis of the data shows that these conclusions are not statistically validated. To obtain statistically validated conclusions, data processing was considered in two variants: (i) applying inappropriate data removal tests and (ii) using in statistical analysis the best data between the 25% and 75% quartiles, corresponding to the best results in the middle range of variation.

Before calculating the mean glycemic values and the values of standard deviations for each time point and each product consumed, multiple tests were used to eliminate inappropriate data, namely the box plot test, Grubbs, Chauvenet, Nalimov, Romanowski, Irvin, Q, and Dean-Dixon (considering the statistical probability level of *p* < 0.05). When calculating the means and standard deviations, the values that passed the eight tests at the same time were considered. The results obtained are presented in Table S3. As a result of narrowing the ranges of variation by eliminating the extreme values, two statistically validated situations were highlighted: (i) in the case of WRC intake, blood glucose was attributed the highest value after 30 min, and this value is significantly different from the corresponding blood glucose values at the beginning (t = 0 min) and at intervals of 90 and 120 min; (ii) in the case of Croco crackers consumption, the blood glucose level after 30 min was significantly higher compared to the average blood glucose level after 120 min. Table S4 shows the mean glycemic values and the corresponding values of standard deviations, calculated based on the best 50% of the individual values corresponding to each case (time and type of product consumed). By narrowing down the range of variation, additional statistically validated conclusions were drawn: (i) blood glucose levels present peaks half an hour after consumption for all product types consumed. These values exceed all other times considered and are statistically significant (*p* < 0.05); (ii) there are no statistically significant differences between blood glucose values at baseline (t = 0), after 90 and 120 min (*p* > 0.05); (iii) when consuming WRC and Croco crackers, blood glucose levels after 60 min are significantly lower than the corresponding blood glucose levels after 30 min; (iv) during similar times, there are no statistically significant differences between the three cases corresponding to the products consumed, except for the case recorded at 120 min, for which the lowest blood glucose level for Croco crackers consumption was identified.

Another approach to variations in blood glucose over time, depending on the product consumed, was to analyze the differences between means from baseline (Table S5). Compared to the case where all average blood glucose levels were analyzed (Table S2), where no conclusion was reached, in this case it is highlighted that in the case of Croco crackers consumption, the blood glucose levels at 90 and 120 min are significantly lower in comparison to the blood sugar levels after 30 min. As shown in Figure 4b, the highest blood glucose levels for all consumed products are recorded after 30 min, with no statistically significant differences between the three products consumed. In this case, too, additional options for statistical data processing were considered when analyzing the increase in blood sugar fluctuations compared to the baseline measurements (t = 0 min). After removing irrelevant data, the mean blood glucose values are shown in Table S6, and the mean glycemic data is displayed in Table S7, considering every single value that falls within the interquartile range. In this case, when conducting an analysis of blood sugar variation relative to baseline (t = 0 min), the following additional (statistically significant) information was obtained: (i) all blood sugar values 30 min after the consumption of the three products were higher compared to the blood sugar levels after 60, 90, and 120 min; (ii) after 60 min, the lowest average blood glucose value was recorded during Croco crackers consumption, which is clearly different from the other two studied cases; (iii) after 120 min, Croco crackers generated the lowest average blood glucose value, which is significantly different from the similar cases of consuming WRC and BRC. The lower effect of WRC and BRC on postprandial blood glucose and insulin response may be due to a higher amount of amylopectin long chain than usual rice varieties [32]. Compared to Croco crackers, fluctuations in blood glucose levels are not statistically significant; (iv) there are no statistically significant differences in blood glucose variations between WRC and BRC intake.

The glycemic peaks are recorded 30 min after consuming the three products, followed by a drop in the blood glucose levels. The highest peaks in blood sugar levels (after 30 min) correspond to the consumption of Croco crackers. The greatest drops in blood glucose levels occur when Croco crackers are consumed after the glycemic spike has been reached (postprandial hypoglycemia). These results indicate that, for a short-term increase in blood glucose levels (corresponding to the increase in energy levels during exercise), Croco crackers can be successfully used as a quick energy delivery product, but there is a possibility of postprandial hypoglycemia 90–120 min after administration. When consuming WRC and BRC, the increase in blood glucose level 30 min after consumption is lower than in the case of consuming Croco crackers. Decreased postprandial glucose and insulin response is a beneficial health effect for both healthy individuals and those with impaired carbohydrate metabolism [32].

There are no statistically significant differences (*p* > 0.05) in the glycemic response between the two types of rice flour crackers. In Japanese clinical practice, rice cracker snacking has also been shown to increase postprandial blood glucose levels, which can make it difficult to maintain ideal blood sugar control [57]. The work of Watanabe et al. [58] suggests that switching from white rice crackers to black rice crackers suppresses the increase in blood glucose levels.

### 3.8. Sensory Analysis

**Figure 5:**
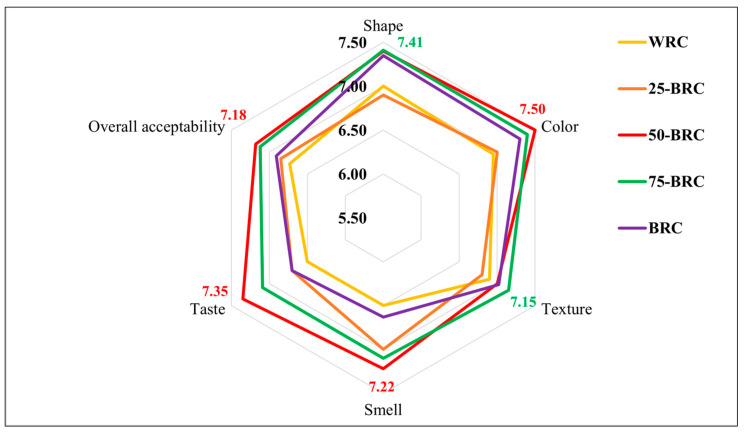
The results of the sensory analysis related to the crackers made of whole brown rice flour, black rice flour, and their mixtures: WRC-crackers made of 100% whole brown rice flour, BRC-crackers made of 100% whole black rice flour, 25-BRC-crackers made of 25% black rice flour and 75% whole brown rice flour, 50-BRC-crackers made of 50% whole brown rice flour and 50% black rice flour, and 75-BRC-crackers made of 75% whole brown rice flour and 25% black rice flour.

The decisive influence of the addition of BRF on the sensory properties of the examined crackers is revealed in Figure 5.

Regarding shape, the results of the sensory analysis revealed the highest score (7.41) for 75-BRC. The panelists positively rated the “attractive” dark color of the crackers with up to 100% increased BRF content, with an average score of 7.30. Surprisingly, WRC was rated with the lowest color score (7.00) compared to the darker samples. From a texture perspective, all the samples were scored between 6.80 and 7.15, but without statistical significance. When comparing BRC and WRC crackers, some panelists commented on the “crispiness” of the samples. The lack of gluten in these products may have prevented the development of a texture similar to that of wheat-based crackers, familiar to many consumers. The panelists judged the smell of the crackers as relatively accurate, as it appeared “neutral”. One of the most important characteristics, taste, was highly valued in all samples and described by panelists as “earthy” and “spicy”, particularly in the darker cracker samples. However, the highest score (7.35) was achieved by 50-BRC. The mean overall acceptability scores ranged from 6.73 to 7.18, with a strong positive relationship to taste acceptability (r = 0.86), with the highest score (7.18) being attributed to 50-BRC, followed by 75-BRC (7.12). The crackers made entirely from brown rice flour (WRC) received the lowest rating of 6.73 and were attributed the lowest consumer acceptability.

## 4. Conclusions

This study investigated the possibility of using black rice flour to improve the nutritional and physicochemical properties of gluten-free crackers. This is the first research focused on the characterization and analysis of gluten-free crackers made from mixtures of whole brown and black rice flour. The findings demonstrated that, in a ratio-dependent manner, the addition of BRF to WRF improved the proximate composition of the samples by raising the content of protein, crude fiber, lipids, polyphenols, proanthocyanidins, flavonoids, and total minerals.

The CIEL a*b* color parameters showed different trends within the examined samples. Both the surface and the crushed samples experienced a decrease in lightness and yellowness and an increase in redness upon the addition of BRF. The characteristic brown color of the surface layer was attributed to the formation of Maillard reaction products. The reduction in the hue values of a* and b* in the crust, along with the rise in the ratio of BRF, indicated an intense hydroxylation process of the B-rings in the anthocyanins. ΔΕ values increased as the amount of brown rice flour was substituted. The addition of BRF lowered the spread ratio of the crackers but had no influence on the textural parameters of the samples.

The sensory analysis revealed that 50% of BRC received the highest score in terms of “overall acceptability”. The samples entirely made of WRF and BRF were not positively accepted by the consumers due to their “spicy” and “earthy” taste. The crispiness of the BRF-based crackers was positively evaluated by the consumers. The findings of the study suggested that the substitution of WRF with BRF in proportion to 50% can result in a nutritionally improved gluten-free snack that is widely accepted by consumers.

The results of glycemic variation analyses confirmed that consumption of crackers made from WRF or BRF does not generate glycemic peaks above the 30 mg/dL limit from baseline blood glucose levels, suggesting that crackers made from whole rice grains can also be consumed by people with type 2 diabetes mellitus as an alternative to regular biscuit snacks.

Referencing data for the gluten-free bakery industry, the results of this study can be used to develop better nutritional quality for similar rice-based food products.

## Figures and Tables

**Table 1:** Formulation and coding of dough and corresponding crackers.

Raw Material	Cracker Code (Black Rice Flour Ratio *)				
	WRC	25-BRC	50-BRC	75-BRC	BRC
Whole brown rice flour, g	122.5	91.90	61.25	30.63	-
Whole black rice flour, g	-	30.6	61.25	91.87	122.5
Whey protein, g	12.5	12.5	12.5	12.5	12.5
Xanthan gum, g	3	3	3	3	3
Margarine, g	30	30	30	30	30
Sugar, g	1.25	1.25	1.25	1.25	1.25
Salt, g	3	3	3	3	3
Baking powder, g	3.74	3.74	3.74	3.74	3.74
Water (distilled), g	65	65	65	65	65

* WRC—crackers made of 100% whole brown rice flour; BRC—crackers made of 100% black rice flour; 25-BRC—crackers made of 25% black rice flour and 75% whole brown rice flour; 50-BRC—crackers made of 50% black rice flour and 50% whole brown rice flour; 75-BRC—crackers made of 75% black rice flour and 25% whole brown rice flour.

**Table 2:** Characteristics of the investigated rice crackers.

Parameter	WRC	25-BRC	50-BRC	75-BRC	BRC	“Croco” Crackers
Moisture, %	5.55 ± 0.27 ^b^	5.20 ± 0.16 ^b^	5.60 ± 0.26 ^b^	5.40 ± 0.09 ^b^	6.19 ± 0.16 ^a^	-
aw	0.38 ± 0.04 ^a^	0.34 ± 0.16 ^a^	0.28 ± 0.07 ^a^	0.37 ± 0.01 ^a^	0.42 ± 0.02 ^a^	-
Protein, g/100 g	9.54 ± 0.19 ^c^	10.11 ± 0.25 ^bc^	10.68 ± 0.22 ^ab^	11.16 ± 0.34 ^a^	11.44 ± 0.46 ^a^	8.30
Total lipids, g/100 g	12.70 ± 0.26 ^b^	13.04 ± 0.40 ^ab^	13.28 ± 0.34 ^ab^	13.44 ± 0.43 ^ab^	13.90 ± 0.30 ^a^	25.40
Ash, g/100 g	4.38 ± 0.02 ^b^	4.33 ± 0.14 ^b^	4.32 ± 0.10 ^b^	4.15 ± 0.09 ^b^	4.64 ± 0.08 ^a^	-
Crude fiber, g/100 g	2.51 ± 0.30 ^e^	2.88 ± 0.05 ^d^	3.25 ± 0.15 ^c^	3.61 ± 0.14 ^b^	3.98 ± 0.08 ^a^	2.60
Total starch, %	40.54 ± 1.49 ^a^	39.95 ± 1.13 ^a^	39.34 ± 1.27 ^a^	38.75 ± 0.83 ^a^	38.14 ± 0.90 ^a^	-
Carbohydrates, g/100 g	70.87 ± 2.59 ^a^	69.64 ± 2.14 ^ab^	68.47 ± 1.78 ^ab^	67.64 ± 1.15 ^ab^	66.04 ± 1.93 ^b^	60.00
Energy, Kcal/100 g	440.96 ± 12.25 ^a^	442.12 ± 13.10 ^a^	442.62 ± 12.98 ^a^	443.38 ± 14.35 ^a^	442.98 ± 11.09 ^a^	508.00

Results are presented as mean values ± standard deviations (*n* ≥ 3); Different letters within the same row indicate significant differences (*p* < 0.05) between mean values (Tukey test); WRC—crackers made of 100% whole brown rice flour; BRC—crackers made of 100% black rice flour, 25-BRC—crackers made of 25% black rice flour and 75% whole brown rice flour; 50-BRC—crackers made of 50% black rice flour and 50% whole brown rice flour; 75-BRC—crackers made of 75% black rice flour and 25% whole brown rice flour; Data on the proximate analysis of “Croco” crackers was provided by the manufacturer.

**Table 3:** Colorimetric analysis of investigated cracker doughs and resulting rice crackers.

CIEL* a* b* Parameters in Dough	**Dough** **Containing 100% Whole Brown Rice Flour (WRC)**	**Dough** **Containing 25% Black Rice Flour** **(25-BRC)**	**Dough** **Containing 50% Black Rice Flour** **(50-BRC)**	**Dough** **Containing 75% Black Rice Flour** **(75-BRC)**	**Dough Containing 100% Whole Black Rice Flour** **(BRC)**
L*	75.43 ± 1.24 **^a^**	46.98 ± 0.36 **^b^**	36.57 ± 1.73 **^c^**	31.58 ± 0.07 **^d^**	26.07 ± 1.12 **^e^**
a*	0.39 ± 0.16 **^c^**	5.83 ± 0.24 **^a^**	5.84 ± 0.09 **^a^**	5.83 ± 0.21 **^a^**	4.79 ± 0.74 **^b^**
b*	19.45 ± 0.49 **^a^**	3.65 ± 0.02 **^b^**	1.35 ± 0.33 **^c^**	1.25 ± 0.39 **^c^**	−0.03 ± 0.06 **^d^**
Corresponding color (RGB coordinates)					
CIEL* a* b* Parameters on the Cracker Surface	**Crackers Containing 100% Whole Brown Rice Flour (WRC)**	**Crackers Containing 25% Black Rice Flour** **(25-BRC)**	**Crackers Containing 50% Black Rice Flour** **(50-BRC)**	**Crackers Containing 75% Black Rice Flour** **(75-BRC)**	**Crackers Containing 100% Black Rice Flour (BRC)**
L*	70.75 ± 0.52 **^a^**	43.65 ± 0.40 **^b^**	36.10 ± 1.07 **^c^**	30.35 ± 2.40 **^d^**	27.90 ± 0.10 **^d^**
a*	1.83 ± 0.18 **^d^**	6.56 ± 0.18 **^a^**	6.62 ± 0.27 **^a^**	5.76 ± 0.47 **^b^**	4.95 ± 0.20 **^c^**
b*	26.04 ± 0.41 **^a^**	8.16 ± 0.09 **^b^**	5.13 ± 1.17 **^c^**	2.59 ± 0.52 **^d^**	1.55 ± 0.09 **^d^**
∆E on the surface	-	32.81 ± 0.34 **^c^**	40.75 ± 0.94 **^b^**	46.88 ± 1.90 **^a^**	49.45 ± 0.53 **^a^**
Browning index (BI)	46.62 ± 0.51 **^a^**	31.29 ± 0.74 **^b^**	28.24 ± 0.28 **^c^**	22.22 ± 0.59 **^d^**	18.13 ± 0.08 **^e^**
Chroma (C*)	26.30 ± 0.45 **^a^**	10.47 ± 0.20 **^b^**	8.38 ± 0.35 **^c^**	5.76 ± 0.13 **^d^**	5.19 ± 0.22 **^d^**
Corresponding color (RGB coordinates)					
CIEL* a* b* Parameters in the Crushed Sample	**Crackers Containing 100% Whole Brown Rice Flour (WRC)**	**Crackers Containing 25% Black Rice Flour (25-BRC)**	**Crackers Containing 50% Black Rice Flour (50-BRC)**	**Crackers Containing 75% Black Rice Flour** **(75-BRC)**	**Crackers Containing 100% Whole Black Rice Flour (BRC)**
L*	76.58 ± 0.21 **^a^**	46.81 ± 0.83 **^b^**	35.89 ± 0.66 **^c^**	30.84 ± 0.82 **^d^**	26.35 ± 0.36 **^e^**
a*	2.20 ± 0.08 **^c^**	9.71 ± 0.22 **^b^**	10.75 ± 0.05 **^a^**	10.97 ± 0.18 **^a^**	9.77 ± 0.18 **^b^**
b*	27.94 ± 0.26 **^a^**	12.19 ± 0.16 **^b^**	8.65 ± 0.47 **^c^**	6.47 ± 0.06 **^d^**	5.37 ± 0.37 **^e^**
∆E in the crushed sample	-	34.49 ± 0.64 **^d^**	45.84 ± 0.50 **^c^**	51.28 ± 0.65 **^b^**	55.59 ± 0.21 **^a^**
Browning index (BI)	46.36 ± 0.31 **^b^**	44.84 ± 0.43 **^c^**	48.67 ± 0.88 **^a^**	48.36 ± 0.53 **^a^**	48.59 ± 0.47 **^a^**
Chroma (C*)	28.03 ± 0.27 **^a^**	15.58 ± 0.27 **^b^**	13.80 ± 0.47 **^c^**	12.74 ± 0.19 **^d^**	11.15 ± 0.41 **^e^**
Corresponding color (RGB coordinates)					

Results are presented as mean values ± standard deviations (*n* ≥ 3); Different letters within the same row indicate significant differences (*p* < 0.05) between mean values (Tukey test).

**Table 4:** Textural properties of investigated rice crackers.

Parameter		WRC	25-BRC	50-BRC	75-BRC	BRC
Medium width, mm		35.30 ± 0.39 ^a^	35.60 ± 0.16 ^a^	35.00 ± 0.54 ^a^	34.80 ± 0.35 ^a^	35.20 ± 0.19 ^a^
Medium thickness, mm		4.70 ± 0.05 ^d^	5.10 ± 0.10 ^c^	5.10 ± 0.21 ^c^	5.40 ± 0.04 ^b^	5.70 ± 0.12 ^a^
Spread factor		75.3 ± 2.20 ^a^	69.30 ± 3.40 ^b^	68.10 ± 1.50 ^b^	64.0 ± 1.10 ^bc^	61.50 ± 2.30 ^c^
Texture parameters	Hardness (N)	39.88 ± 9.49 ^a^	39.80 ± 9.70 ^a^	42.08 ± 7.84 ^a^	40.85 ± 7.34 ^a^	42.61 ± 7.25 ^a^
	Peak count (−)	8.70 ± 3.31 ^a^	12.05 ± 3.56 ^a^	12.25 ± 3.96 ^a^	13.55 ± 3.78 ^a^	12.90 ± 3.77 ^a^
	Work of shear (Ns)	66.30 ± 16.93 ^a^	65.87 ± 15.84 ^a^	67.08 ± 14.27 ^a^	79.80 ± 13.82 ^a^	86.17 ± 15.72 ^a^
	Distance to max. peak (mm)	1.21 ± 0.27 ^a^	1.36 ± 0.27 ^a^	1.40 ± 0.26 ^a^	1.36 ± 0.24 ^a^	1.49 ± 0.22 ^a^

Results are presented as mean values ± standard deviations (*n* ≥ 3); Different letters within the same row indicate significant differences (*p* < 0.05) between mean values (Tukey test); WRC—crackers made of 100% whole brown rice flour; BRC—crackers made of 100% black rice flour; 25-BRC—crackers made of 25% black rice flour and 75% whole brown rice flour; 50-BRC—crackers made of 50% black rice flour and 50% whole brown rice flour; 75-BRC—crackers made of 75% black rice flour and 25% whole brown rice flour.

**Table 5:** Total phenolic compounds of investigated rice crackers.

Parameter		WRC	25-BRC	50-BRC	75-BRC	BRC
Polyphenols, mg GAE/100 g of sample	Free polyphenols	53.14 ± 2.37 ^d^	60.18 ± 2.15 ^cd^	67.20 ± 2.03 ^bc^	74.23 ± 3.11 ^ab^	81.25 ± 4.07 ^a^
	Bound polyphenols	31.18 ± 1.03 ^e^	53.30 ± 1.15 ^d^	75.43 ± 3.02 ^c^	97.56 ± 3.74 ^b^	119.69 ± 5.91 ^a^
	Total polyphenols	84.32 ± 3.69 ^e^	113.48 ± 4.82 ^d^	142.63 ± 4.29 ^c^	171.79 ± 6.33 ^b^	200.94 ± 9.83 ^a^
Total flavonoids (TFV), µg/g		309.31 ± 22.18 ^e^	606.44 ± 28.94 ^d^	924.60 ± 19.11 ^c^	1278.60 ± 43.16 ^b^	2592.07 ± 89.49 ^a^
Total proanthocyanidins (TPA), µg/g		38.58 ± 0.29 ^e^	234.48 ± 3.29 ^d^	422.14 ± 5.10 ^c^	549.73 ± 5.10 ^b^	854.75 ± 17.21 ^a^

Results are presented as mean values ± standard deviations (*n* ≥ 3); Different letters within the same row indicate significant differences (*p* < 0.05) between mean values (Tukey test); WRC—crackers made of 100% whole brown rice flour; BRC—crackers made of 100% black rice flour; 25-BRC—crackers made of 25% black rice flour and 75% whole brown rice flour; 50-BRC—crackers made of 50% black rice flour and 50% whole brown rice flour; 75-BRC—crackers made of 75% black rice flour and 25% whole brown rice flour.

**Table 6:** U-HPLC analysis of anthocyanins and phenolic acids in rice flour crackers.

Anthocyanins, µg/g of Sample	Sample Code				
	WRC	25-BRC	50-BRC	75-BRC	BRC
Cyanidin 3-O-glucoside chloride	nd	42.33 ± 1.55 ^d^	92.29 ± 3.47 ^c^	130.60 ± 4.21 ^b^	221.58 ± 2.07 ^a^
Peonidin-3-O-glucoside chloride	nd	23.27 ± 0.68 ^d^	50.93 ± 1.76 ^c^	72.15 ± 4.32 ^b^	120.25 ± 5.21 ^a^
Oenin chloride	nd	0.54 ± 0.09 ^c^	0.87 ± 0.08 ^b^	1.98 ± 0.08 ^a^	1.86 ± 0.03 ^a^
Petunidin chloride	nd	0.18 ± 0.02 ^d^	0.31 ± 0.03 ^c^	0.36 ± 0.01 ^b^	0.44 ± 0.01 ^a^
Myrtillin chloride	nd	nd	nd	nd	nd
Callistephin chloride	nd	nd	nd	nd	nd
**Phenolic Acids, µg/g of sample**	**WRC**	**25-BRC**	**50-BRC**	**75-BRC**	**BRC**
3,4-dihydroxybenzoic acid	nd	62.00 ± 0.09 ^c^	109.55 ± 5.56 ^c^	171.52 ± 2.35 ^b^	223.41 ± 1.28 ^a^
Vanillic acid	0.32 ± 0.03 ^e^	8.12 ± 0.10 ^d^	14.99 ± 0.72 ^c^	22.81 ± 0.01 ^b^	31.17 ± 1.04 ^a^
Syringic acid	3.21 ± 0.13 ^c^	2.52 ± 0.09 ^d^	2.70 ± 0.21 ^d^	4.13 ± 0.02 ^b^	7.79 ± 0.23 ^a^
p-coumaric acid	1.45 ± 0.12 ^a^	1.39 ± 0.06 ^a^	1.31 ± 0.01 ^a^	1.25 ± 0.03 ^a^	0.98 ± 0.16 ^b^
Ferulic acid	6.15 ± 0.16 ^a^	5.90 ± 0.11 ^ab^	5.89 ± 0.03 ^ab^	6.15 ± 0.16 ^a^	5.58 ± 0.12 ^c^
Sinapic acid	0.62 ± 0.06 ^a^	0.53 ± 0.07 ^ab^	0.46 ± 0.02 ^bc^	0.37 ± 0.01 ^cd^	0.29 ± 0.04 ^d^
Ellagic acid	0.24 ± 0.01 ^e^	2.91 ± 0.05 ^d^	5.29 ± 0.24 ^c^	8.95 ± 0.29 ^b^	11.84 ± 0.18 ^a^
p-hydroxybenzoic acid	nd	nd	nd	nd	nd
Procyanidin B2	nd	nd	nd	nd	nd
Gallocatechin gallate	nd	nd	nd	nd	nd

WRC—crackers made of 100% whole brown rice flour; BRC—crackers made of 100% black rice flour; 25-BRC—crackers made of 25% black rice flour and 75% whole brown rice flour; 50-BRC—crackers made of 50% black rice flour and 50% whole brown rice flour; 75-BRC—crackers made of 75% black rice flour and 25% whole brown rice flour; nd—not detected; Results are presented as mean values ± standard deviations (*n* ≥ 3); Different letters within the same row indicate significant differences (*p* < 0.05) between mean values (Tukey test).

## Data Availability

The original contributions presented in the study are included in the article and Supplementary Materials, further inquiries can be directed to the corresponding author.

## Supplementary Materials

The following supporting information can be downloaded at: https://www.mdpi.com/article/10.3390/foods13101503/s1, Table S1. Formulation and coding of dough and corresponding crackers; Table S2. Presentation of average blood glucose values; Table S3. Presentation of average blood glucose levels after elimination of extremes; Table S4. Presentation of mean glycemic values considering data corresponding to the interquartile domain; Table S5. Increases in average blood glucose values from baseline; Table S6. Increases in mean blood glucose levels after elimination of extremes; Table S7. Increases in mean glycemic values considering data corresponding to the interquartile range.
