# Supplementary material for: Whole Black Rice Flour Improves the Physicochemical, Glycemic, and Sensory Properties of Cracker Snacks
Source: Foods. 2024 May 13;13(10):1503. doi: 10.3390/foods13101503 (PMC11120445; doi:10.3390/foods13101503)
Supplement: Supplementary file 1 [file foods-13-01503-s001.zip › foods-2977234-supplementary.pdf]

Appendix A – Supplementary Material

Section 2. Materials and methods

Section 2.2. Preparation of rice crackers

Table S1. Formulation and coding of dough and corresponding crackers.

Raw Material	Cracker code (Black rice flour ratio*)									
	WRC		25-BRC		50-BRC		75-BRC		BRC	
	Basic dough	Fatty dough	Basic dough	Fatty dough	Basic dough	Fatty dough	Basic dough	Fatty dough	Basic dough	Fatty dough
**Whole brown rice flour, g**	100	22.5	75	16.9	50	11.25	25	5.63	-	-
**Whole black rice flour, g**	-	-	25	5.6	50	11.25	75	16.87	100	22.5
**Whey protein, g**	10	2.5	10	2.5	10	2.5	10	2.5	10	2.5
**Xanthan gum, g**	3	-	3	-	3	-	3	-	3	-
**Margarine, g**	15	15	15	15	15	15	15	15	15	15
**Sugar, g**	1.25	-	1.25	-	1.25	-	1.25	-	1.25	-
**Salt, g**	1	2	1	2	1	2	1	2	1	2
**Baking powder, g**	1.87	1.87	1.87	1.87	1.87	1.87	1.87	1.87	1.87	1.87
**Water (distilled), g**	65	-	65	-	65	-	65	-	65	-

* WRC – crackers made of 100% whole brown rice flour, BRC – crackers made of 100% black rice flour, 25-BRC – crackers made of 25% black rice flour and 75% whole brown rice flour, 50-BRC – crackers made of 50% black rice flour and 50% whole brown rice flour, 75-BRF – crackers made of 75% black rice flour and 25% whole brown rice flour;

2.3. Dry matter content

Drying the homogenized samples at 105°C to constant weight allowed for the measurement of the dry matter content using the gravimetric method (Binder GmbH, Germany).

2.4 Water activity analysis

Using a portable water activity analyzer (Rotronic HygroPalm, Switzerland), the water activity (a_w) was ascertained. A predetermined amount of the sample was crushed and put in a plastic vat in order to conduct the analysis. After 12 minutes, the result was read out from the analyser sensor, which was set on top of a sampling vessel that held the vat containing the sample for analysis. Every sample was subjected to a triplicate analysis.

2.5. Total proteins content

To quantify the protein content, the Kjeldahl assay was employed (Velp Scientifica UDK 127, Italy) **[Error! Bookmark not defined.]** and the results were calculated according to the Eq. 1.SM:

$$Protein\ (g/100\,g\ on\ dry\ weight\ basis) = TN * 6.25 \quad \text{(Eq.1.SM)}$$

where TN represents the total nitrogen, %, and 6.25 is the conversion factor of nitrogen into protein.

2.6. Total lipids content

Soxhlet extraction was performed for the determination of the total lipid content (Velp Scientifica 148, Italy) **[Error! Bookmark not defined.]**, and the results were expressed as g of lipids/100g of dry sample.

2.7. Ash content

The samples were introduced in a muffle furnace and incinerated for 24 hours at 550 ± 10°C (Nabertherm Germany) **[Error! Bookmark not defined.]**, and the ash level was expressed as g of ash/100g of dry sample.

2.8. Crude fiber

The procedure established by the Commission Regulation (EC) No. 152/2009 **[Error! Bookmark not defined.]** was employed to quantify the amount of crude fiber, and the results were calculated according to Eq. 2.SM:

$$Crude\ fiber\ (g/100\ g) = (m_0 - m_1) * 100/m \quad \text{(Eq. 2.SM)}$$

where m is the weight of the sample, g; m_0 is the loss of weight after washing, g; and m_1 stands for the loss of weight after ashing during the blank test, g.

Section 3. Results and discussions

Section 3.7. Impact of the consumption of whole rice flour-based crackers on postprandial blood glucose levels

Table S2. Presentation of average blood glucose values

Raw data (all data on domain 0 – 120 minutes)					
**Average**	**0 min**	**30 min**	**60 min**	**90 min**	**120 min**
1 (WRC)	101.75 ± 12.46 (a1)	125.08 ± 10.25 (a1)	115.08 ± 13.61 (a1)	105.76 ± 8.40 (a1)	103.28 ± 10.21 (a1)
2 (BRC)	100.58 ± 13.27 (a1)	118.57 ± 12.43 (a1)	112.54 ± 14.90 (a1)	101.28 ± 12.32 (a1)	101.32 ± 9.69 (a1)
3 (Croco biscuits)	99.63 ± 14.61 (a1)	129.58 ± 21.87 (a1)	119.77 ± 18.73 (a1)	108.41 ± 13.44 (a1)	98.68 ± 13.58 (a1)

*On lines, average values with different letters are statistically significantly different ($p < 0.05$);
*On columns, average values with different figures are statistically significantly different ($p < 0.05$).
*WRC – crackers made of 100% whole brown rice flour, BRC – crackers made of 100% black rice flour

Table S3. Presentation of average blood glucose levels after elimination of extremes

Removal of inappropriate data (all data on domain 0 – 120 minutes)					
**Average**	**0 min**	**30 min**	**60 min**	**90 min**	**120 min**
1 (WRC)	101.75 ± 12.46 (a1)	121.53 ± 8.14 (a1)	110.74 ± 11.68 (a1)	102.45 ± 7.58 (a1)	101.13 ± 8.41 (a1)
2 (BRC)	100.58 ± 13.27 (a1)	120.87 ± 10.24 (a1)	110.52 ± 11.57 (a1)	101.34 ± 10.18 (a1)	100.08 ± 8.77 (a1)
3 (Croco biscuits)	99.63 ± 14.61 (a1)	126.43 ± 18.43 (a1)	111.50 ± 15.36 (a1)	104.51 ± 11.08 (a1)	99.28 ± 12.47 (a1)

*On lines, average values with different letters are statistically significantly different ($p < 0.05$);
*On columns, average values with different figures are statistically significantly different ($p < 0.05$);
*WRC – crackers made of 100% whole brown rice flour, BRC – crackers made of 100% black rice flour

Table S4. Presentation of mean glycemic values considering data corresponding to the interquartile domain

Data between Q (75) and Q (25) (all data on domain 0 – 120 minutes)					
**Average**	**0 min**	**30 min**	**60 min**	**90 min**	**120 min**
1 (WRC)	101.58 ± 4.88 (c1)	124.09 ± 3.97 (a1)	114.36 ± 4.09 (ab1)	106.87 ± 2.58 (bc1)	103.59 ± 2.61 (c1)
2 (BRC)	99.58 ± 5.02 (b1)	118.47 ± 4.67 (a1)	109.24 ± 3.28 (ab1)	101.22 ± 6.49 (b1)	100.68 ± 2.82 (b1)
3 (Croco biscuits)	99.94 ± 3.66 (c1)	125.04 ± 2.39 (a1)	111.55 ± 4.55 (b1)	103.28 ± 4.20 (bc1)	99.68 ± 3.04 (c1)

*On lines, average values with different letters are statistically significantly different ($p < 0.05$);

*On columns, average values with different figures are statistically significantly different ($p < 0.05$);
*WRC – crackers made of 100% whole brown rice flour, BRC – crackers made of 100% black rice flour

Table S5. Increases in average blood glucose values from baseline

Raw data (variations on the interval of 30-120 minutes as compared to 0 minutes)					
**Average**	**0 min**	**30 min**	**60 min**	**90 min**	**120 min**
1 (WRC)		23.33 ± 9.74 (a1)	13.33 ± 11.25 (a1)	4.01 ± 10.08 (a1)	1.53 ± 7.09 (a1)
2 (BRC)		17.99 ± 7.27 (a1)	11.96 ± 9.58 (a1)	0.70 ± 12.95 (a1)	0.74 ± 13.48 (a1)
3 (Croco biscuits)		29.95 ± 13.28 (a1)	20.14 ± 16.53 (a1)	8.78 ± 12.44 (a1)	-0.95 ± 13.35 (a1)

Table S6. Increases in mean blood glucose levels after elimination of extremes

Remove inappropriate data (variations on the interval of 30-120 minutes as compared to 0 minutes)					
**Average**	**0 min**	**30 min**	**60 min**	**90 min**	**120 min**
1 (WRC)		22.87 ± 8.71 (a1)	13.62 ± 10.23 (a1)	4.29 ± 9.87 (a1)	1.53 ± 6.52 (a1)
2 (BRC)		17.99 ± 7.27 (a1)	11.82 ± 9.06 (a1)	0.84 ± 11.25 (a1)	0.70 ± 12.84 (a1)
3 (Croco biscuits)		28.34 ± 13.07 (a1)	21.15 ± 14.44 (a1)	8.78 ± 12.44 (a1)	-0.87 ± 12.81 (a1)

Table S7. Increases in mean glycemic values considering data corresponding to the interquartile range

Data between Q (75) and Q (25) (variations on the interval of 30-120 minutes as compared to 0 minutes)					
**Average**	**0 min**	**30 min**	**60 min**	**90 min**	**120 min**
1 (WRC)		21.43 ± 5.49 (a1)	13.58 ± 6.30 (ab1)	4.39 ± 4.25 (b12)	1.53 ± 0.73 (b1)
2 (BRC)		17.99 ± 7.27 (a1)	11.76 ± 2.68 (ab1)	0.88 ± 3.21 (b12)	0.73 ± 5.48 (b12)
3 (Croco biscuits)		27.85 ± 6.04 (a1)	21.26 ± 4.29 (ab1)	8.50 ± 5.05 (bc1)	-0.93 ± 7.31 (c23)
